# Emergence of resistant dermatophytosis caused by *Trichophyton indotineae*: First case series in Thailand

**DOI:** 10.1016/j.mmcr.2025.100713

**Published:** 2025-06-07

**Authors:** Sutsarun Prunglumpoo, Kunyanut Krongboon, Klaichan intarachaieua, Anzawa Kazushi, Thareena Bunnag, Wattamon Paunrat, Wanchanida komkhong

**Affiliations:** a Institute of Dermatology, Bangkok, Thailand; b Kanazawa Medical University Hospital, Japan

**Keywords:** *Trichophyton indotineae*, Recalcitrant dermatophytosis, *Trichophyton mentagrophyte* complex, Resistant dermatophytosis, Urease test

## Abstract

We identified 5 Thai patients diagnosed with *Trichophyton indotineae* infection after screening of patients with culture results consistent with *Trichophyton mentagrophytes* complex who had negative urease test and confirmed by molecular analysis in DNA sequencing at the internal transcribed spacer region, including abnormalities in the Squalene epoxidase gene in 2 patients, which confirmed the spread of terbinafine resistant dermatophytosis in Thailand.

## Graphical abstract

**Figure:**
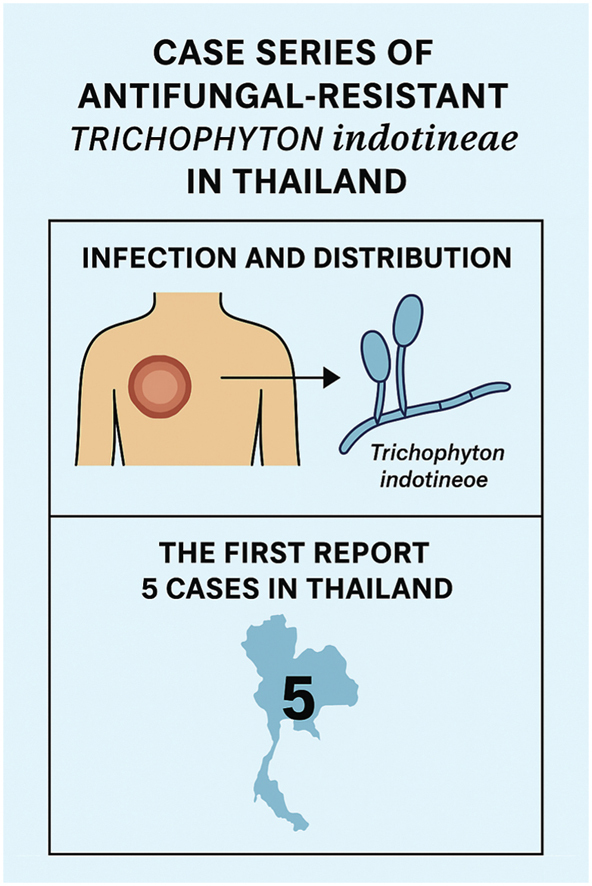

Dermatophytosis is a common superficial fungal infection worldwide. infection usually affects the skin, Scalp, Or nails and is most commonly caused by organisms of *Trichophyton*, *Microsporum*, Or *Epidermophyton*[1,2]

In 2018, it was reported in India that a large-scale emergence of terbinafine-resistant Trichophyton interdigitalis [3] was detected. Then, in 2020, Kano reported the organism's presence in Japan, which was renamed *Trichophyton indotineae* [4].

In addition, there have been reports of the spread of *Trichophyton indotineae* in many countries, such as Asia, the USA, Latin America, and Europe. Still, there has never been a report of infection in Thailand before. Only a German patient with a history of residing in Thailand and India was found to have tested positive in Germany [5].

The diagnosis of *Trichophyton indotineae* infection is based on molecular analysis, which involves performing DNA sequencing at the internal transcribed spacer regions. However, some reports [4,6] found that a urease test can help differentiate *Trichophyton indotineae* from *Trichophyton interdigitale* and *Trichophyton mentagrophyte*.

We therefore began to screen for *Trichophyton Indotineae* infection by screening colony specimens with morphology consistent with the *Trichophyton mentagrophyte* complex with a urease test and diagnosing the organism with Molecular analysis, including the squalene epoxidase gene mutation test and the terbinafine antifungal susceptibility test.

## 1 The study

Our study screened 30 colony specimens from January 2024 to April 2024 at the Institute of Dermatology, Thailand, using the urease test, similar to a previous report [4,6]. These specimens had macroscopic and microscopic features compatible with the *Trichophyton mentagrophyte* complex. Of these, 7 (28 %) had negative urease test results. We used *Trichophyton mentagrophyte* IOD67155 and *Trichophyton rubrum* IOD67721 as positive and negative control strains for the urease test.

**Fig. 1:**
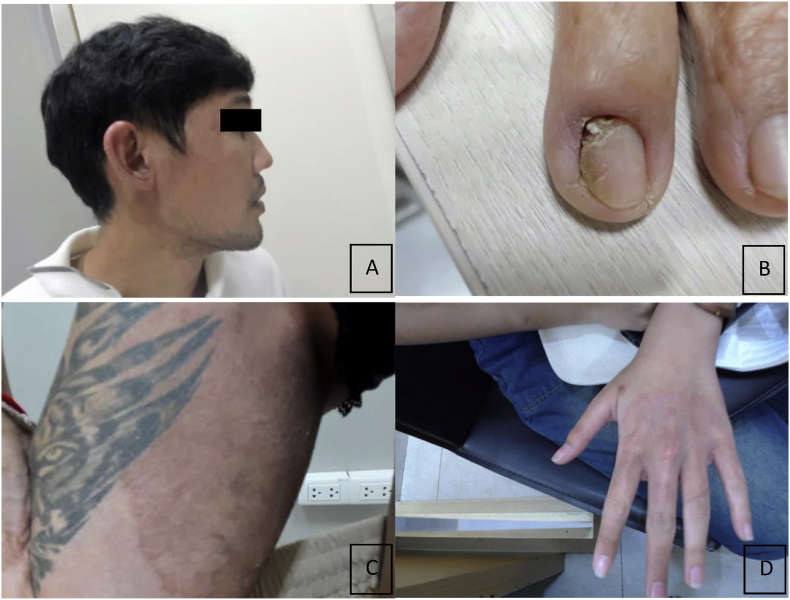
The image illustrates the clinical presentation of the patient's cutaneous lesions. Figure A: Patient No.1 presented with a chronic annular patch on the face. Figure B: Patient No.2 presented with distolateral onychomycosis on the fingernail. Figure C: Patient No.3 presented with an extensive circular rash on the groin. Figure D: Patient No.5 presented with a large circular rash on the dorsal surface of the hand.

When clinically analyzed, the five patients, as shown in Table 1, were all in Thailand. There was no history of traveling abroad or contact with patients with dermatophyte fungi. The patients were two males and three females aged 18–81 years. Most of them presented with rashes distributed in multiple locations, as shown in Fig. 1, especially on the buttocks, groin, or trunk. 3 patients presented with rashes on the face (Tinea facialis), and one presented with Tinea unguium. All patients had a history of drug resistance and had been treated with various oral antifungal drugs. Three patients had a history of being treated with terbinafine, but their symptoms did not improve.

**Table 1:** Clinical manifestation and clinical outcome of 5 patients with resistant dermatophytosis caused by *Trichophyton indotineae*.

Patient no.	Age/Sex/Nationality	Underlying disease	Clinical	Onset	Prior Treatment	Responsive treatment	Urease test	DNA sequencing	SQLE gene	MIC of TBF mg/liter
1	29/M/Thailand	No	Tinea facialis , Tinea Corporis	1 year	Griseofulvin 500 mg/day 6 weeks	Itraconazole 200 mg/day 1 month	Negative	*Trichophyton indotineae*	Wild type	0.5
2	81/F Thailand	HT	Tinea unguium	3 mths	Topical treatment	Itraconazole pulse 100 2 × 2 mg x 2 pulse	Negative	*Trichophyton indotineae*	Wild type	0.5
3	25/F Thailand	HIV + ve CD822	Tinea corporis, Tinea cruris Tinea facialis	1 year	Terbinafine 250 mg 10 weeks	Itraconazole 200 mg/day 2 months	Negative	*Trichophyton indotineae*	Phe397Leu 1191 C > A	>1
4	65/M Thailand	No	Tinea corporis, Tinea cruris	1 year	Griseofulvin 500 mg/day 4 weeks, Terbinafine 250 mg/day 4weeks, Fluconazole 200 mg/week 4 weeks	Itraconazole 200 mg/day 3 months	Negative	*Trichophyton indotineae*	Wild type	1
5	18/F Thailand	No	Tinea facialis , Tinea manuum	3 mths	Griseofulvin 500 mg/day 4 weeks, Terbinafine 250 mg/day 4weeks	Itraconazole 200 mg/day 1 month	Negative	*Trichophyton indotineae*	Phe397Leu 1189 T > C	>1

All five isolates were diagnosed by molecular analysis, using DNA sequencing of the internal transcribed spacer region using ITS1 and ITS4 primers. The PCR products were subjected to nucleotide blast sequencing and were compatible with all 5 *Trichophyton indotineae* isolates, compared with the *Trichophyton indotineae* LC508728 reference sequences [6]. In addition, we also subjected all isolates to squalene epoxidase gene mutation studies using primers FW3 and RV3, as referenced [7], by amplifying the SQLE gene portion of the DNA extract. The PCR products were then sequenced to determine the nature of the point mutation. It was found that 2 out of 5 isolates (40 %) had SQLE gene mutation, located at position Phe397Leu in two instances (1191C > A in 1 case and 1189T > C in 1 case), compared with the reference sequence from GenBank accession numbers MW188000 and MW187980 [7]. In addition, we tested the MIC of the isolates with terbinafine. The broth microdilution method was performed following the CLSI guideline, and it was found that 2 in 5 isolates had high MIC values > 1 mg/L, like as shown in Table 1. *Trichophyton mentagrophyte* ATCC MYA-4439 was used as a quality control strain in this method [8].

## 2 Discussion

Dermatophytosis is a significant and growing problem worldwide. Before the 2018 report of terbinafine-resistant dermatophytosis [3] in India, there were some reports of drug resistance in *Trichophyton rubrum* [9] or *Trichophyton mentagrophyte* [10,11], which was believed to be due to the combination of antibiotics and steroids used to treat the fungus, which caused mutations [12]. It is now known that the pathogen is *Trichophyton indotineae* or *Trichophyton mentagrophytes* ITS genotype VIII, which has a mutation in the SQLE gene that disrupts the function of the enzyme squalene epoxidase. As a result, terbinafine cannot inhibit the synthesis of the fungal cell membrane. However, a report [7] found that missense mutations can occur in several locations. However, the most common mutation found in 53.13 % was found at position 1189T > C, 1191C > A, or 1191C > G, resulting in Phe397Leu, with the 1189T > C trait (35.9 %) being the most common, while in our case, abnormalities were found in 2 out of 5 isolates (40 %), with the trait of 1191C > A in 1 case and 1189T > C in 1 case, both of which had a history of actual resistance to terbinafine.

However, in the report [7], it was found that isolates of *Trichophyton indotineae* with some gene mutations had low MIC values to terbinafine, such as position 1342G > A, which caused Ala448Thr to have an MIC value of 0.0625. This may be consistent with some reports that *Trichophyton indotineae* isolates were sensitive to terbinafine [13].

In addition, it has been reported [14] that *Trichophyton indotineae* has mutations in other genes, such as mutations in the ERG11/CYP51 gene. This gene controls the upregulation of ABC transporters, which are involved in the action of azole drugs.

Diagnosis of *Trichophyton indotineae* still requires molecular analysis because we cannot differentiate the species by macroscopic or microscopic features. DNA sequencing of the internal transcribed spacer region may still be the gold standard, along with an antifungal susceptibility test for terbinafine and searching for the AMR gene (antimicrobial resistance gene) to help diagnose the organism. However, these techniques may still be complicated in some countries or small hospitals. Due to the organism's properties, the urease test, which is inexpensive and easy to obtain, can help distinguish *Trichophyton indotineae* (Urease test negative) from other organisms in the *Trichophyton mentagrophyte* complex (Urease test positive) [4,6].

From our study, all five isolates that gave negative urease test results when tested for DNA sequencing at the ITS region were compatible with *Trichophyton indotineae*. The urease test may be another screening test that can help diagnose *Trichophyton indotineae*. However, further studies with larger samples may be required to determine the sensitivity and specificity of the urease test.

There are several reports of treating *Trichophyton indotineae* infection using different drugs. From the study [15], it is recommended to use itraconazole as the first-line drug, with itraconazole (200 mg/day) given for at least 1–3 months. In cases where terbinafine is required, it may be effective (terbinafine sensitive) only in wild-type SQLE or a mutation causing Ala448Thr by giving terbinafine a double dose (500 mg/day) or giving 250 mg/day for 3 months. In our report, most patients responded well to itraconazole.

## 3 Conclusion

We describe five patients diagnosed with *Trichophyton indotineae* for the first time in Thailand. As PCR and DNA sequencing techniques may underdiagnose the disease, and the prevalence of infection in Thailand is expected to be higher, the Urease test, a simple and inexpensive test, may help diagnose the infection.

## CRediT authorship contribution statement

**Sutsarun Prunglumpoo:** Writing – original draft, Visualization, Validation, Project administration, Methodology, Formal analysis, Data curation, Conceptualization. **Kunyanut Krongboon:** Visualization, Validation, Methodology, Investigation, Conceptualization. **Klaichan intarachaieua:** Writing – review & editing, Validation. **Anzawa Kazushi:** Validation. **Thareena Bunnag:** Visualization, Validation, Investigation. **Wattamon Paunrat:** Visualization, Validation, Investigation. **Wanchanida komkhong:** Visualization, Validation, Investigation.

## Ethics statements

The patient's images and information have been obtained with informed consent.

## Ethical statement

I am Dr. Sutsarun prunglumpoo and have studied the data of a sample case of dermatophytosis from *Trichophyton indotineae* in the Institute of Dermatology, Thailand. The patient's images and information have been obtained with informed consent. Confidentiality and integrity of information I will ensure that the results in this case report are reported honestly and without bias. My goal is to contribute to knowledge while maintaining the highest ethical standards.

## Funding

Research did not receive any specific grant from funding agencies in the public, commercial, or not-for-profit sectors.
